# Syndrome de Boerhaave rapidement fatal: une urgence à ne pas méconnaitre

**DOI:** 10.11604/pamj.2013.14.73.2467

**Published:** 2013-02-22

**Authors:** Nawfal Houari, Nabil Kanjaa

**Affiliations:** 1 Service de Réanimation Polyvalente, CHU Hassan II, Fès, Maroc

**Keywords:** Syndrome de Boerhaave, hydropneumothorax, pneumomédiastin, pneumopéricarde, Boerhaave syndrome, hydropneumothorax, pneumomediastinum, pneumopericardium

## Image en medicine

Il s'agit d'un patient de 48 ans, alcoolique chronique, ayant présenté après un repas copieux et des efforts de vomissements une détresse respiratoire. L'examen physique a retrouvé un emphysème sous-cutané cervical chez un patient en état de choc septique. La TDM thoracique a objectivé un hydropneumothorax bilatéral, un pneumomédiastin et un pneumopéricarde. Le patient a bénéficié en urgence d'une oesophagectomie avec jéjunostomie d'alimentation, mais l’évolution était rapidement fatale par un choc septique réfractaire. Le syndrome de Boerhaave est une rupture spontanée de l'œsophage. Il se manifeste par la triade de Meckler associant des vomissements, une douleur rétrosternale et un emphysème sous-cutané cervical. La nature barogénique de la rupture explique probablement la contamination médiastinale large, responsable rapidement d'une détresse respiratoire grave, d'une septicémie et d'un état de choc. Une anamnèse soigneuse et un examen détaillé de l'imagerie permettent un diagnostic rapide. La thoracotomie avec oesophagectomie s'impose en cas de présence de sepsis.

**Figure 1:**
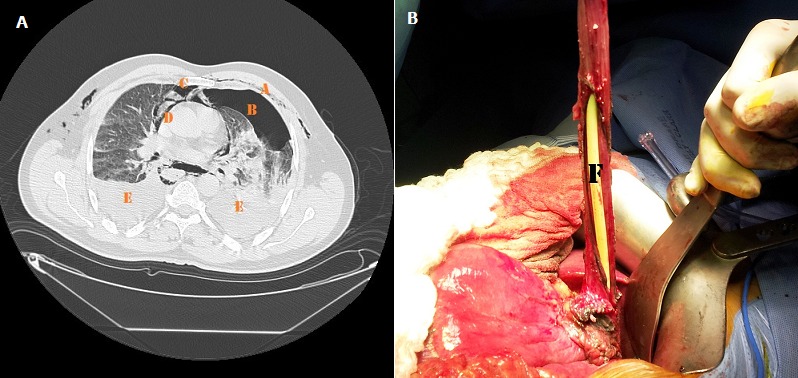
(A) TDM thoracique objectivant: a- Emphysème sous-cutané; b-Pneumothorax; c-Pneumomédiastin; d-Pneumopéricarde; e-Epanchement pleural bilatéral; (B): f-Découverte d'une rupture du tiers inférieur de l'œsophage

